# Expression analysis and single-nucleotide polymorphisms of *SYNDIG1L* and *UNC13C* genes associated with thoracic vertebral numbers in sheep (*Ovis aries*)

**DOI:** 10.5194/aab-64-131-2021

**Published:** 2021-04-23

**Authors:** Ying-Jie Zhong, Yang Yang, Xiang-Yu Wang, Ran Di, Ming-Xing Chu, Qiu-Yue Liu

**Affiliations:** Key Laboratory of Animal Genetics and Breeding and Reproduction of the Ministry of Agriculture and Rural Affairs, Institute of Animal Science, Chinese Academy of Agricultural Sciences, Beijing 100193, P. R. China

## Abstract

The objective of the current study was to analyze expression levels of synapse differentiation inducing 1-like (*SYNDIG1L*) and unc-13 homolog C (*UNC13C*) genes in different tissues, while single-nucleotide polymorphisms (SNPs) of two genes were associated with multiple thoracic vertebrae traits in both Small-tailed Han sheep (STH) and Sunite sheep (SNT). The expression levels of *SYNDIG1L* and *UNC13C* were analyzed in the brain, cerebellum, heart, liver, spleen, lung, kidney, adrenal gland, uterine horn, longissimus muscle, and abdominal adipose tissues of two sheep breeds with different thoracic vertebral number (TVN) sheep (T13 groups and T14 groups) by real-time quantitative polymerase chain reaction (RT-qPCR). Meanwhile, the polymorphisms of *UNC13C* gene g.52919279C>T and *SYNDIG1L* gene g.82573325C>A in T14 and T13 were genotyped by the Sequenom MassARRAY^®^ SNP assay, and association analysis was performed with the TVN. The results demonstrated that *UNC13C* gene was extensively expressed in 11 tissues. The expression of *UNC13C* gene in longissimus muscle of T14 groups of STH was significantly higher than that of T13 groups (P<0.05). *SYNDIG1L* gene was overexpressed in brain and cerebellum tissues, and the expression level of *UNC13C* gene in the brain and cerebellum of T13 groups in SNT was significantly higher than that of T14 groups (P<0.01). Association analysis showed that SNPs found in the *UNC13C* gene had no significant effects on TVN for both two genes. The polymorphism of *SYNDIG1L* g.82573325C>A was significantly correlated with the TVN in both STH (P<0.05) and SNT (P<0.01). Taken together, the *SYNDIG1L* gene was related to thoracic vertebral development, and this variation may be potentially used as a molecular marker to select the multiple thoracic vertebrae in sheep.

## 1 Introduction

The spine of a vertebrate consists of a series of repeated vertebrae. Based on morphological differences, the vertebrae were subdivided into five distinct functional spinal regions: cervical, thoracic, lumbar, sacral, and caudal (Donaldson et al., 2013). The number of vertebrae is relatively conserved among mammalian species. However, the quantitative variations of vertebrae (Sun et al., 2019) have been observed in pigs (Rohrer and Nonneman, 2017), deer (Mizer and Wahl, 2018), humans (Ibrahim et al., 2013), and sheep (Donaldson et al., 2013). In general, the vertebrae of sheep were arranged from the neck to the sacral part according to 7 cervical vertebrae (C), 13 thoracic vertebrae (T), 6 lumbar vertebrae (L), and 4 sacral vertebrae (S), with a total of 30 vertebrae. Among them, mutations in the thoracolumbar position were the most common (T14L6 or T13L7) (Zhang, 1996). Multi-vertebrae sheep have advantages in adaptability and meat production performance (Zhang et al., 1996). The cultivation of multi-spine sheep has comprehensive benefits for the economy, society, and ecology. This is of great significance to improve the quality and efficiency of the animal husbandry industry.

Among domestic animals, the most extensive studies of vertebral number variation have focused on pigs. Previous studies have reported quantitative trait loci (QTL) for vertebral numbers in pigs by genome scans based on microsatellite markers. Two genome-wide significant QTLs were detected on pig chromosomes (SSCs) 1 and 2 in a Meishan and Göttingen cross line (Wada et al., 2000). The vertebrae-development-associated (VRTN) gene on the SSC7 and NR6A1 gene on SSC1 were considered as candidate genes affecting vertebral numbers. Fine mapping of vertebral number trait was performed, and an orphan nuclear receptor, germ cell nuclear factor (NR6A1) was localized to be the candidate and also confirmed by multiple studies (Mikawa et al., 2011; Zhang et al., 2015), which was confirmed in various studies (Fan et al., 2013; Rohrer et al., 2015; Yang et al., 2016). However, current studies on sheep vertebral number traits were superficial, and functional studies focused on genomic variations were relatively rare (Cao et al., 2015; Chen et al., 2012).

Small-tailed Han sheep (STH) selected in this study is one of the famous indigenous sheep breeds of China which grows fast and has good early puberty and high fecundity (Guo et al., 2020). Sunite sheep (SNT) are also an indigenous breed that has the advantages of cold/drought resistance, fast growth, and good disease resistance. Meat production performance is good, lean meat percentage is high, protein content is high, it has a high popularity, and it has a large demand space at home and even abroad (Zhong et al., 2020; Gao et al., 2014). There was a high proportion of multiple thoracic and lumbar vertebral numbers in both sheep breeds. Identification of molecular markers of multi-spine variation for marker-assisted selection is of great significance for the improvement of meat production performance.

Previously, we conducted a genome-wide association analysis in two sheep breeds of 670 sheep with different thoracic vertebra numbers using a Affymetrix ovine 600K single-nucleotide polymorphism (SNP) array. Genome-wide significant associations were detected at nine SNPs in the 245 kb region with $p<1.13\times 10^{-9}$ (Yingjie Zhong, unpublished data). The significant SNPs on chromosome 7 were located near the region of synapse differentiation inducing 1-like (*SYNDIG1L*) and unc-13 homolog C (*UNC13C*). Whole-genome resequencing was also performed on 40 sheep with thoracic vertebra numbers for fine mapping. Using the top 10 % of $F_{\mathrm{st}}$ values as cutoffs, candidate genes associated with thoracic vertebra number were identified, which included *SYNDIG1L* and *UNC13C* genes. Annotation of the sheep reference genome (Oar4.0) suggested that the two non-synonymous mutations are located in protein-coding regions of synapse differentiation inducing 1-like *SYNDIG1L* and *UNC13C* genes respectively. g.82573325C>A located on exon 3 of *SYNDIG1L* is a non-synonymous mutation that changes amino acid position 186 from glycine (G) to tryptophan (W), while *UNC13C* g.52919279C>T located on exon 14 is also a non-synonymous mutation that changes amino acid position 1465 from valine (V) to isoleucine (I).

The purpose of this study is to explore the association of *UNC13C* g.52919279C>T and *SYNDIG1L* g.82573325C>A loci with thoracic vertebral number. It provides promising candidate causal mutations for further research on the number of vertebral variations on sheep.

## 2 Materials and methods

### 2.1 Animal and main reagent

All the experimental procedures mentioned in the present study were approved by the Science Research Department (in charge of animal welfare issues) of the Institute of Animal Science, Chinese Academy of Agricultural Sciences (IAS-CAAS) (Beijing, China). In addition, there was ethics approval by the animal ethics committee of IAS-CAAS (no. IAS2020-82, 28 July 2020).

A total of 12 healthy ewes aged 3 years old were selected from the livestock and breeding base of Tianjin Animal Husbandry and Veterinary Research Institute. The number of thoracic vertebrae of SNT and STH was 13 and 14, respectively. After slaughter, 11 tissues of brain, cerebellum, heart, liver, spleen, lung, kidney, adrenal gland, uterine horn, longissimus muscle, and abdominal fat were quickly collected, put into a 2 mL RNase-free centrifuge tube, and stored in liquid nitrogen immediately. After returning to the laboratory, they were stored in the freezer at -80 ${}^{\circ }$C.

For genotyping (Table 1), a total of 383 sheep were selected from SNT and STH from Bayan Nur slaughterhouses in the Inner Mongolia autonomous region, China, and Yuncheng slaughterhouses in Shandong province. After slaughter, the collected fresh muscle tissue was quickly put into a 2 mL frozen storage tube and immediately stored in liquid nitrogen. After being brought back to the laboratory, all the fresh muscle tissue was transferred to a freezer at -80 ${}^{\circ }$C for storage.

**Table 1:** Sample information of the real-time quantitative polymerase chain reaction (RT-qPCR) and genotyping.

Breed	Thoracic	Tissue	RT-qPCR	Genotyping
	vertebral no.		sample no.	sample no.
SNT	13	Brain, cerebellum, heart, liver,	3	122
	14	spleen, lung, kidney, adrenal,	3	66
STH	13	uterine horn, longissimus muscle,	3	137
	14	abdominal fat	3	58
Total			12	383

### 2.2 Extraction of genomic DNA and total RNA and main reagents

DNA from muscle tissue was extracted by a DNA extraction kit (TIANGEN Biotech, Beijing, China). The total RNA of tissue was extracted using the Trizol and Qiagen RNeasy kit (Qiagen). The concentration and integrity of DNA and RNA were detected by Nanodrop2000, and the quality of DNA and RNA was detected by 1.5 % agarose gel electrophoresis.

Quantitative polymerase chain reaction (PCR) was done using the SYBR Green fluorescent dye for product detection (SYBR^®^ Premix Ex *Taq*™ II). The PrimeScript™ RT reagent kit was used to synthesize cDNA (TaKaRa, Beijing).

### 2.3 cDNA synthesis

The total volume of the reaction system was 20 µL / 4.0 µL 5× PrimeScript buffer (for real time), 1.0 µL PrimeScript RT enzyme mix E, 1.0 µL Oligo dT primer, 1.0 µL random 6 mers, 1000 ng RNA. The remaining system was supplemented with RNase-free ddH${}_{2}$O. The reaction condition of PCR was 37 ${}^{\circ }$C for 15 min and 85 ${}^{\circ }$C for 5 s. The product obtained after reverse transcription was diluted five times and stored in a freezer at -20 ${}^{\circ }$C for detection of tissue expression of the target gene.

### 2.4 Primer design

Primers for real-time quantitative polymerase chain reaction (RT-qPCR) were designed using the GenBank database (https://www.ncbi.nlm.nih.gov/genbank, last access: 10 July 2020). Genes and their accession numbers include *SYNDIG1L* (GenBank: XM_027972017.1), *UNC13C* (GenBank: XM_027971817.1). The β *-actin* (GenBank: NM_001009784.2) was an internal reference gene. The primers (Table 2) were synthesized by Beijing Tianyi Huiyuan Biotechnology Co., Ltd.

**Table 2:** The primer information for RT-qPCR.

Name	Primer sequence	Product	$T_{m}$
		size (bp)	(${}^{\circ }$C)
*SYNDIG1L*	F: TCTCCCAGTGACCAGCAAGG	133	60
	R: GCCACCACCACGGCTACAT		
*UNC13C*	F: CAAACCTCACAGAGTCGCCC	198	60
	R: CTTGTCTCCGAGGTTGGGTC		
β*-actin*	F: CCAACCGTGAGAAGATGACC	97	60
	R: CCCGAGGCGTACAGGGACAG		

### 2.5 Real-time fluorescent quantitative PCR

Using a Roche Light Cycler^®^ 480 type II fluorescence quantitative PCR instrument, the whole PCR process was monitored in real time by fluorescence signal accumulation, and β *-actin* was used as an internal reference gene. The total volume of the reaction system was 20 µL: SYBR Premix Ex Taq II 10 µL, forward primer 0.8 µL, reverse primer 0.8 µL, RNase-free ddH${}_{2}$O 6.4 µL, and cDNA 2.0 µL. PCR conditions were as follows: initial denaturation at 95 ${}^{\circ }$C for 5 s, followed by 40 cycles of 95 ${}^{\circ }$C for 10 s and 60 ${}^{\circ }$C for 30 s.

### 2.6 Genotyping

Genotyping of *UNC13C* g.52919279C>T and *SYNDIG1L* g.82573325C>A was carried out using the Sequenom MassARRAY^®^ SNP (Johansen et al., 2013; Ortega et al., 2017) assay. The primer information is provided in Table 3. The typing sample is DNA, and the amount required for each sample is 20 µL. DNA concentration ranged from 40 to 80 ng/ µL.

**Table 3:** Primer sequences for genotyping.

Name	Primer sequence (5${}^{\prime }$–3${}^{\prime }$)
*SYNDIG1L* g.82573325C>A	F: ACGTTGGATGCGTGCAGAGCAGAAGCCCT
	R: ACGTTGGATGATCTTCTCCATGCTCTGCTG
	E: CCTTCTACTTCTCCCAG
*UNC13C* g.52919279C>T	F: ACGTTGGATGAGCAGCAAATCGATCTGAGG
	R: ACGTTGGATGTGTAATGAGCACCTTGCTGG
	E: TCGATCTGAGGCAGAAA

F: upstream primer; R: downstream primer; E: extension primer.

### 2.7 Statistical analysis

The relative expressions of *UNC13C* and *SYNDIG1L* were calculated by the 2${}^{-\Delta \Delta \mathrm{Ct}}$ method. The difference of relative expression between the T13 group and the T14 group was analyzed by one-way ANOVA. The allele frequency, genotype frequency, p value, polymorphism information content (PIC), heterozygosity ($H_{e}$), and effective allele number ($N_{e}$) were calculated using Microsoft Excel 2016 statistical software. Then, the distribution of genotypes for each SNP in the studied populations was tested for deviation from Hardy–Weinberg equilibrium. P>0.05 indicates the locus was under Hardy–Weinberg equilibrium.

The correlation between SNP and thoracic vertebra number traits of two varieties was analyzed by SAS (V.9.4) (SAS Institute Inc.). The p values < 0.05 were considered to be significant. The mathematical models are as follows: Fisher's exact probability test and logistic regression.

$$\begin{array}{rl} & \text{Logistic regression model:}\\ & \;\;\;\mathrm{Logit}\;(y)=\mathrm{ln}\left(\frac{y}{1-y}\right)=\beta_{0}+\beta_{1}X_{1}+\beta_{2}X_{2}+\ldots \\ & +\beta_{n}X_{n}+e \end{array}$$

Here, y represents the number of thoracic vertebrae, $X_{1}$ represents the variety, and $X_{2}$ represents the genotype.

## 3 Results

### 3.1 Polymorphism analysis of *SYNDIG1L* and *UNC13C* genes

**Figure 1:**
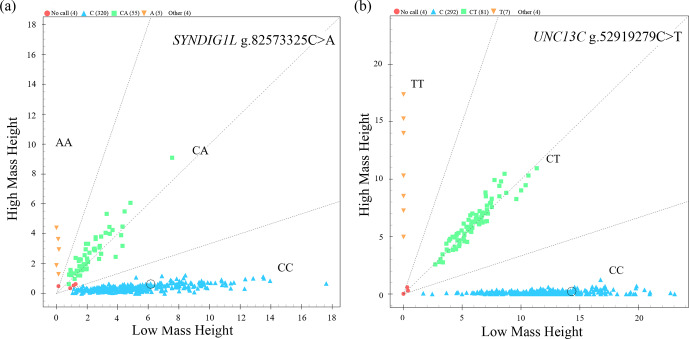
Genotyping results. **(a)** Scatter plot of *SYNDIG1L* genotyping results. **(b)** Scatter plot of *UNC3C* genotyping results.

The genotyping results of 383 sheep (Fig. 1) showed that two candidate loci were polymorphic (Table 4). *UNC13C* g.52919279C>T and *SYNDIG1L* g.82573325C>A displayed low polymorphisms (*PIC* < 0.25) in both SNT and STH populations. Statistical significance was analyzed by the chi-square test. The frequency of *SYNDIG1L* g.82573325C>A was consistent with Hardy–Weinberg equilibrium in SNT, ensuring the reliability of their application to evaluating larger groups (P>0.05). *SYNDIG1L* g.82573325C>A was, however, under Hardy–Weinberg imbalance (P<0.05) in the STH population. *UNC13C* g.52919279C>T satisfied the Hardy–Weinberg equilibrium in both populations (P>0.05).

**Table 4:** Population genetic analysis of candidate loci in two sheep breeds.

Gene	SNP	Breed	Sample			Total	Genotype			Gene		PIC	$H_{e}$	$N_{e}$	p
			size				frequency			frequency					
			TT	CT	CC		TT	CT	CC	T	C				
*UNC13C*	g.52919279C>T	SNT	4	38	143	185	0.02	0.21	0.77	0.12	0.88	0.19	0.22	1.28	0.441
		STH	3	43	149	195	0.02	0.22	0.76	0.13	0.87	0.20	0.22	1.28	0.959
			AA	CA	CC		AA	CA	CC	A	C				
*SYNDIG1L*	g.82573325C>A	SNT	1	32	152	185	0.01	0.17	0.82	0.09	0.91	0.15	0.17	1.20	0.620
		STH	4	23	168	195	0.02	0.12	0.86	0.08	0.92	0.14	0.15	1.17	0.007

$H_{e}$: heterozygosity; PIC: polymorphic information content; $N_{e}$: number of effective alleles; P>0.05 indicates the locus was under Hardy–Weinberg equilibrium.

### 3.2 Association analysis of *SYNDIG1L*and *UNC13C* genes with thoracic vertebral number in two breeds

Firstly, association analysis of SNPs with thoracic vertebral number (TVN) was explored in two sheep breeds, respectively. The statistical results were shown in Table 5. *UNC13C* g.52919279C>T had no significant effect on TVN in both the STH and SNT populations (P>0.05). *SYNDIG1L* g.82573325C>A was significantly correlated with different thoracic vertebral numbers in both STH (P<0.05) and SNT (P<0.01). Then, logistic regression was used to test the effects of breeds and genotypes on different thoracic vertebral numbers in sheep, which was shown in Table 6. For the two candidate SNPs, the breeds have no significant relevance with TVN in sheep (P>0.05). The genotypes of *SYNDIG1L* g.82573325C>A were significantly associated with multiple thoracic vertebrae in sheep (P<0.01), indicating that this gene might be correlated with a TVN trait in the sheep.

**Table 5:** Genotypes of candidate locus and the number of thoracic vertebrae in a single breed by Fisher's exact test.

Gene	SNP	Breed	Fisher's exact
			test (p value)
*UNC13C*	g.52919279C>T	SNT	0.8342
		STH	0.6503
*SYNDIG1L*	g.82573325C>A	SNT	0.0048
		STH	0.0199

P≤0.05 indicates the significant difference; P≤0.01 indicates the extremely significant difference.

**Table 6:** Logistic regression for genotypes, breeds, and different thoracic vertebral numbers.

Gene	SNP	Breed	Genotype
		(p value)	(p value)
*UNC13C*	g.52919279C>T	0.2763	0.9706
*SYNDIG1L*	g.82573325C>A	0.2408	0.0023

P≤0.05 indicates the significant difference; P≤0.01 indicates the extremely significant difference.

### 3.3 Expression profiles of *UNC13C* and *SYNDIG1L* genes in SNT and STH with different TVNs

**Figure 2:**
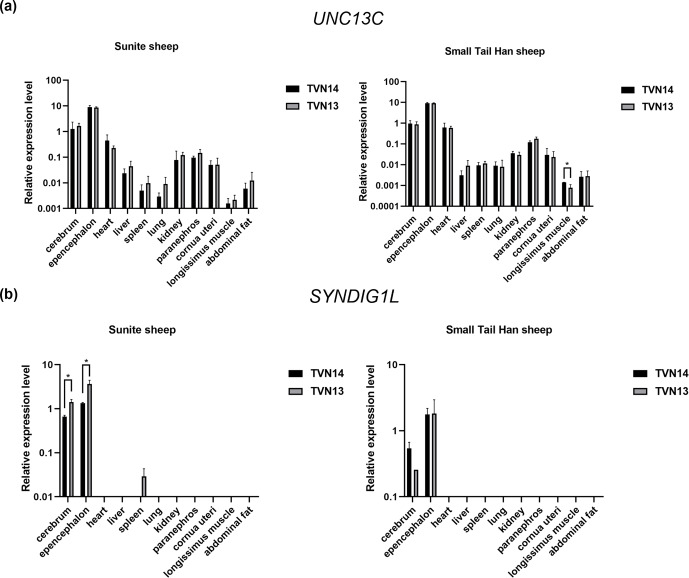
Results of expression level of *UNC13C* and *SYNDIG1L* genes in STH and SNT with different TVNs. **(a)** The comparison of expression levels of *UNC3C* between SNT and STH. **(b)** The comparison of expression levels of *SYNDIG1L* between SNT and STH. The significant results with a p value lower than 0.05 are given one asterisk (∗).

The results of RT-qPCR showed that the *UNC13C* gene was extensively expressed in 11 tissues of STH and SNT (Fig. 2a). The *SYNDIG1L* gene was mainly expressed in brain tissue and slightly expressed in the spleen in SNT with T13 (Fig. 2b). The *UNC13C* gene was highly expressed in the cerebellum of STH. The expression of *UNC13C* in group T14STH was significantly higher than group T13STH in the longissimus muscle (P<0.05); the gene expression fold change is 1.8. In T13SNT, the expression of the *SYNDIG1L* gene in the brain and cerebellum tissues was significantly higher than that in T14SNT (P<0.05); the fold changes of gene expression were 0.5 and 0.4, respectively.

## 4 Discussion

The vertebrae of mammals are derived from the mesoderm of the gastrula. Vertebrae development is an extremely complicated system that is regulated temporally and spatially. It has been known that any error in development can result in many congenital abnormalities (Gilbert, 2003). Zhang et al. (1998) found that the meat-production performance of multi-vertebrae sheep was significantly better than that of normal sheep, with longer longissimus muscle, larger abdominal cavity volume, carcass weight, net meat weight, lean meat percentage, and other economic indexes. Moreover, this trait is heritable. Thus, it is important to understand the mechanism of vertebral number variation from the molecular level and apply it to sheep breeding with multiple thoracic vertebrae. The genetic architecture of thoracic vertebral number has been extensively studied in pigs, and major genes affecting this trait have been mapped in indigenous pigs (Rohrer et al., 2015; Duan et al., 2018; Liu et al., 2020). However, current studies on sheep vertebral numbers were superficial, and functional studies focused on genomic variations were relatively rare.

Liu et al. (2020) found that regulation variants on SSC7 might play crucial roles in the number of thoracic vertebrae (NTV) and the *FOS* (Fos proto-oncogene, AP-1 transcription factor subunit) on SSC7, and *BMPR1A* was identified as a novel candidate gene affecting the NTV in pigs on SSC14. Fan et al. (2013) identified three loci for a vertebral number trait through a genome-wide association study and located them in a 947 kb region on SSC7 in pigs. The locus was refined to 100 kb by a homologous sharing test, which contained only *VRTN* and *SYNDIG1L* genes. Among them, *VRTN* is considered to be the main candidate gene affecting vertebral numbers in the modern western world. The *VRTN* gene was considered as a candidate gene affecting vertebral number also in sheep (Li et al., 2019). We believe that it is not accidental that *SYNDIG1L* and *VRTN* have been identified at the same time. *SYNDIG1L* is highly expressed in the striatum (de Chaldée et al., 2006). This is consistent with our previous RT-qPCR results. As one of the basal ganglia of the brain, the striatum is mainly responsible for regulating muscle tension and coordinating various fine and complicated movements (Lorenc-Koci et al., 1998; Hemsley and Crocker, 2001). Meanwhile, it is related to the occurrence of Parkinson's disease (Miyanishi et al., 2019; Choe et al., 2011), chorea (Ishikawa et al., 1990), and other diseases. Correspondingly, some researchers found that the incidence of dyskinesia increased with the increase of thoracic vertebral numbers in pigs (Nakano et al., 2015). This may be due to the negative effects of high expression of *SYNDIG1L*. On the other hand, *SYNDIG1L* was reported to be a factor affecting the final body weight and back-fat thickness in Landrace pigs (Lee et al., 2018). An et al. (2020) believe that it is the key gene that affects the formation of bovine body shape. We speculate that *SYNDIG1L* may participate in the spine formation process and cause mutation in *SYNDIG1L*, which may lead to abnormal development of vertebrae in sheep. However, more evidence is needed to prove our hypothesis.

The new role of the *UNC13C* gene in oral squamous cell carcinoma (OSCC) has been revealed for the first time. *UNC13C* is a novel tumor suppressor and can be used as a target to prevent oral cancer metastasis (Velmurugan et al., 2019). Studies have shown that *UNC13C* is involved in Alzheimer's disease (AD), which involves dysfunction of many cellular pathways, including synaptic transmission, cytoskeleton dynamics, energetics, and apoptosis (Miller et al., 2013). According to references, *UNC13C* is significantly downregulated in spinal cord tissue of patients with amyotrophic lateral sclerosis (D'Erchia et al., 2017). It is considered to be negatively correlated with muscle ability in the study of myasthenia (Hangelbroek et al., 2016), no direct relationship between the function of the *UNC13C* gene and the number of thoracic vertebrae was found.

## 5 Conclusion

This study found that the polymorphisms of *SYNDIG1L* g.82573325C>A were significantly associated with the thoracic vertebral number in sheep, indicating that this locus may be a promising candidate causal variation in the regulation of thoracic vertebral numbers. Further exploration of the functions of the *SYNDIG1L* gene was necessary for the cultivation of sheep breeds with multiple thoracic vertebrae.

## Data Availability

The data sets are available upon request from the corresponding authors.
